# Runner’s Perceptions of Reasons to Quit Running: Influence of Gender, Age and Running-Related Characteristics

**DOI:** 10.3390/ijerph17176046

**Published:** 2020-08-20

**Authors:** Daphne Menheere, Mark Janssen, Mathias Funk, Erik van der Spek, Carine Lallemand, Steven Vos

**Affiliations:** 1 Department of Industrial Design, Eindhoven University of Technology, 5600 MB Eindhoven, The Netherlands; mark.janssen@fontys.nl (M.J.); m.funk@tue.nl (M.F.); e.v.d.spek@tue.nl (E.v.d.S.); c.e.lallemand@tue.nl (C.L.); s.vos@tue.nl (S.V.); 2 School of Sport Studies, Fontys University of Applied Sciences, 5644 HZ Eindhoven, The Netherlands; 3 HCI Research Group, University of Luxembourg, Esch-sur-Alzette, 4365 Luxembourg, Luxembourg

**Keywords:** running drop-out, novice runners, gender, age, running habits, attitudes, interests, motives

## Abstract

Physical inactivity has become a major public health concern and, consequently, the awareness of striving for a healthy lifestyle has increased. As a result, the popularity of recreational sports, such as running, has increased. Running is known for its low threshold to start and its attractiveness for a heterogeneous group of people. Yet, one can still observe high drop-out rates among (novice) runners. To understand the reasons for drop-out as perceived by runners, we investigate potential reasons to quit running among short distance runners (5 km and 10 km) (n = 898). Data used in this study were drawn from the standardized online Eindhoven Running Survey 2016 (ERS16). Binary logistic regressions were used to investigate the relation between reasons to quit running and different variables like socio-demographic variables, running habits and attitudes, interests, and opinions (AIOs) on running. Our results indicate that, not only people of different gender and age show significant differences in perceived reasons to quit running, also running habits, (e.g., running context and frequency) and AIOs are related to perceived reasons to quit running too. With insights into these related variables, potential drop-out reasons could help health professionals in understanding and lowering drop-out rates among recreational runners.

## 1. Introduction

Physical inactivity has become a major public health concern as it is associated with the development of chronic diseases [1,2]. Consequently, the awareness and importance of striving for an active and healthy lifestyle within our society have increased [3]. This is notably reflected in the increased popularity of unorganized recreational sports such as running [4,5]. Running is known for its low threshold to start: it is relatively inexpensive and easy to practice [6] and is associated with many health benefits (i.e., musculoskeletal and cardiovascular health, body composition, and psychological state) [7,8,9,10,11,12,13,14] and is therefore a popular recreational sport. This popularity is especially apparent in the increasing number of commercial running events, and their growing number of participants. In terms of event participation, running is even one of the most popular recreational sports in the world [15,16]. Therefore, since the begin of the 21st century, we can speak of the second wave of running [15].

The growing number and diversity of specialized running events (e.g., ladies runs, color runs, survival runs) are aligned with the development of the heterogeneous profile of ‘the runner’ over the years [17,18,19]. During the first wave of running starting in the 1960s, running used to be dominated by young males [20,21] as it was considered outrageous for women to engage in running [22]. It was not until almost 25 years later, the first Olympic marathon for women was introduced [15]. This partake of women in running continued to develop, where a strong growth is notably visible during the second wave of running, resulting in an almost equal distribution of men and women in recent years [15,19,23]. Similar to data of other Western countries [15], 11.3% of women and 13.2% of men within Dutch adults (i.e., the context of the present study), between the ages 20–79 years, expressed to run at least monthly in 2012 [23], also indicating the age diversity of running participants [15,24]. Besides some socio-demographic characteristics (i.e., gender and age) representing the heterogeneous population of runners, studies showed a variety in terms of motives to partake in running (e.g., health, social and competition elements, performance) [4,25,26]. Furthermore, one can also observe a broader range of different experienced runners (e.g., recreational, competitive) [27] but also running context (e.g., small groups, running partner, individually) [4,19,28]. This diverse profile of ‘the runner’ illustrates that running can appeal to many people (regardless of age, gender, motives, experience or running context) and illustrates the potential of making running even more accessible for an even larger group of people.

Despite the increasing popularity and the growing heterogeneity in runners, one can observe high drop-out rates due to running-related injuries and motivational loss, which is often noticeable among novice runners [29,30,31]. What type of runners are affected by running-related injuries and how this affects a potential drop-out, and how long this drop-out lasts, has been studied extensively in previous literature [29,30,31,32,33]. Although there is evidence on motivations to partake in running [17,25,34,35], reasons to quit running are rather unexplored.

Previous studies on reasons to start running, show the influence of the different type of characteristics. Indicating the influence of socio-demographic variables (i.e., gender and age), running habits (e.g., experience, frequency, relative performance) and in the runners’ attitudes, interests and opinions (AIOs). In a study of Hanson et al. women seemed to be more motivated by AIOs on weight concern, self-esteem, affiliation and psychological coping compared to men and less by AIOs with regards to competition and goal achievement [36]. This is in line with a study by Deaner et al., indicating men reported higher levels of competitiveness compared to women [37]. Motivational differences in age were investigated by Ogles and Masters, indicating young marathon participants (20–28 years) were more motivated by personal goal achievements, compared to older marathon runners (≥50 years). Furthermore, the older participants were more motivated by weight concerns, life meaning, health orientation and affiliation. Besides gender and age, running experience also impacts AIOs towards running. For example, Forsberg et al. showed that more experienced runners, those who run for more than eight years, were more likely to run for social motives and just ‘for the love of running’. Whereas lesser experienced runners, those who run up to three years were more health orientated.

Although motives for running can influence running drop-out [38,39,40], to the best of our knowledge, there is limited evidence about reasons to quit running. An important step toward expanding the evidence base is to understand the reasons for drop-out as perceived by runners. Hence, the scope of this paper is on the perceived reasons to quit running. Janssen et al. distinguish two groups of perceived reasons to quit running: individual (e.g., time management, injuries) and social (e.g., running partner/trainer quits) [4,41]. These reasons are covered by the items of the Leuven Running Survey 2009 [42] and adapted to event runners. Whether these are related to socio-demographic characteristics as gender and age, as they are for motives to running [17,36,37], or running-related characteristics is, however unknown.

With the present study, we aimed to: (i) gain insights in perceived reasons to quit running, and (ii) how this is affected by socio-demographics (i.e., gender and age) running habits, and AIOs on running.

## 2. Materials and Methods

### 2.1. Study Design and Respondents

The data used in this study were drawn from the Eindhoven Running Survey 2016 (ERS2016). We collected data through an online standardized questionnaire among runners at the Eindhoven Marathon Running Event, which offered races at 5, 10, 21.1 and 42.2 km. For this paper, a sub-dataset was drawn with only those runners that participated in the 5 and 10 km races. These distances were selected because of the heterogeneity of the participants, including both more experienced, less and unexperienced runners. The items used in this questionnaire were directly derived from the standardized questionnaire from previous editions of this event (ERS2014 and ERS2015).

In total, 18,261 runners participated in this event, who agreed upon registration that they could be contacted for research purposes. After finishing the event, all runners received an email with an explanation of the study, informed consent and our guarantee that their data would be processed anonymously. If they agreed upon participation in this study, they could click the link to the online questionnaire. The email contained all needed information and was in line with the ethical principles of the Declaration of Helsinki and the American Psychological Association. Thereby, the Research Board of the Fontys School of Sport Studies was consulted prior to initiation of this study, and approval for the study design was obtained.

Of the 18,261 runners, 3727 runners completed the questionnaire (overall response rate of 20.4%) of which 7.9% in the 5 km and 16.2% in the 10 km run. Since this study focused on the 5 and 10 km distances, the subset used here consists of 898 runners (603 who ran the 10 km and 295 the 5 km). The average age of the runners in the present study was 40.7 years, with the youngest runner at 18 years and the oldest 78 years old. 52.7% per cent of the participants were women (n = 474 runners). These socio-demographic backgrounds are comparable to other running samples in previous large-scale running studies in Western Europe [4,15,43].

### 2.2. Questionnaire

The online questionnaire consisted of three sections. The first section included attitudes, interests, and opinions (AIOs) on running, the second focused on socio-demographics and the last on running habits. The questionnaire is provided in the Supplementary Materials of a previous study of Janssen et al. (File S1, questionnaire ERS2016) [41], in which Figure S1 shows a flowchart of the questionnaire.

The first section of the questionnaire consists of items on running AIOs and was adopted from previous studies [4,19,41,43]. Runners were asked to rate the extent to which they agreed with the items, using a 5-point Likert scale (ranging from 1 = totally disagree, to 5 = totally agree). The second section of the questionnaire includes questions on sociodemographic characteristics. We asked for gender (male/female); age (years); professional status (student/unemployed/employed part-time/employed full-time); and level of education (lower and middle/higher/university). The third section covered running habits included running frequency (number of runs per week) years of running experience (<1 year: novice; 1–5 years: moderately experienced; >5 years: experienced); and preferred running context (individual/with friends/colleagues, small running groups/clubs).

### 2.3. Measurements

#### 2.3.1. Creating Scales of Running AIOs

First, we created scales of the items on running AIOs by replicating the questionnaire used by Janssen et al. [41]. We ran reliability analyses for all scales. Items were assessed (Cronbach’s Alpha’s scores of >0.700 were considered acceptable) and reconsidered whether they substantively contributed to the component or not, and no changes were made. Finally, scales were constructed by calculating the average scores for the reliable items per component, resulting in average scale scores. Table 1 gives an overview of these components (i.e., scales), including the number of items, Cronbach’s Alpha’s and average score (ranging from 1 to 5). Eventually, five AIO-scales were formed and used in this study:

(1) Perceived advantages of running (e.g., ‘running gives me energy’, or ‘running is good for my health’);
(2) Identification with running (e.g., ‘I am proud to be a runner’, or ‘I feel myself to be a real runner’);
(3) Running is a sport that is easy to practice (e.g., ‘I can practice running anytime, anywhere’);
(4) Social motives for quitting (e.g., I would quit running ‘if my trainer quit’ or ‘if my running friends quit’);
(5) Individual motives for quitting (e.g., I would quit running if ‘I got injured’, or if ‘my spare time was decreased’).

#### 2.3.2. Dependent Variables

In this study, we used two dependent variables: *social motives for quitting* and *individual motives for quitting*. As they do not follow a normal distribution, both scales were recoded into binary variables. All scores below the scale average (i.e., M = 1.79) were coded as ‘0 below’ and all scores above the average were coded as ‘1 above’. In this way, we were able to interpret the data relative to the sample and able to see if there are variables that could explain why runners score lower or higher compared to their fellow runners.

#### 2.3.3. Independent Variables

As independent variables, we included three groups of variables: (i) socio-demographic variables; (ii) running habits; and (iii) running AIOs. The socio-demographic characteristics included *gender, age,* and *level of education*. The group of running habits consisted of variables that are directly related to running and which define the level of running involvement: *years of running experience, training frequency* and *running context*. The three-remaining scale on running AIOs perceived *advantages of running, identification with running* and *running as a sport that is easy to practice* complete the list of independent variables. Table 2 gives the descriptive statistics of the sample for the dependent and independent variables.

### 2.4. Analysis

All results were analyzed using SPSS 26.0 (IBM Corp., Armonk, NY, USA). First, descriptive statistics (i.e., mean scores, standard deviations, minimum and maximum values) were collected to provide an overview of the sample structure, and the items and variables used. Second, two binary logistic regression models (method = enter) were created with the two dependent variables: *social motives for quitting* and *individual motives for quitting*. As aforementioned, both scales were recoded into binary variables. *Nagelkerke R*^2^ was used as a measure of goodness of fit. Values between 0.10 and 0.20 were considered as satisfactory and above 0.20 as very satisfactory [44,45]. The different models were tested for multicollinearity, outliers, and leverage points by calculating the variance inflation factors and influence statistics (Cook’s). No problems with the data were found concerning these aspects.

## 3. Results

### 3.1. Descriptive Analysis

First, descriptive analysis shows that the social motives for quitting scores an average of 1.79 (SD = 0.72) on a 5-point Likert scale. From the 853 runners, 390 (45.7%) runners score below the group average, and the remaining 54.3% scores above and perceive relatively more social reasons to quit running. For the individual motives for quitting a mean of 3.33 (SD = 0.78) on a 5-point Likert scale was given. Here, of the 853 runners, 399 (46.8%) runners scored below this relative average, and the remaining 46.8% perceived relatively more individual reasons to quit running. In Table 3, the mean scores on the items that form both scales are presented. If we compare these items, it is clear to see that ‘physical constraints or injuries’ are the most important reason to quit running (M = 4.14 SD = 0.77), followed by item 6; ‘tired of running’ (M = 3.20; SD = 1.05). The items that are related to ‘social motives to quit running’, score the lowest (M = 1.82 or lower).

Second, the results of the binary logistic regression are presented in Table 4. The binary logistic regression with social motives for quitting running as a dependent variable showed significant differences (*p* < 0.05, *p* < 0.01 or *p* < 0.001) for gender, experience with running, running context and on the AIOs towards running, viz. running as a sport that is easy to practice, perceived advantage of running and identification with running. The binary logistic regression with individual motives for quitting running as a dependent variable revealed significant differences for age, education level, experience with running, running frequency and one of the AIOs towards running, viz. identification with running.

### 3.2. Binary Logistic Regression Social Reasons for Quitting

In the model for ‘social motives for quitting running’, female runners were more likely (OR = 1.642; *p* < 0.01) to perceive social motives to quit running than male runners. No effect was found for age and education. With regards to the running habits, runners with more than 5 years of running experience, were less likely (OR = 0.610; *p* < 0.05) to perceive social motives to quit running compared to runners with less than 1 year of running experience. Thereby, runners who run with other runners are more likely to perceive social motives to quit running. Those who run with friends, colleagues and in small groups have an odds ratio of 3.352 (*p* < 0.001) and those who run in clubs have an odds ratio of 4.541 (*p* < 0.01), both compared to runners that participate individually. The third running habit; running frequency did not show significant differences. In the final set of independent variables, significant differences for all included AIOs towards running were found. Those who see running as a sport that is easy to practice (OR = 0.502; *p* < 0.01) and those who perceive advantages of running (OR = 0.314; *p* < 0.01) were less likely to perceive social motives to quit running, whereas runners who identify themselves with running (OR = 1.366; *p* < 0.05) were more likely to perceive social motives to quit running.

### 3.3. Binary Logistic Regression Individual Reasons for Quitting

In the model for individual motives for quitting running, gender was not found to be associated with the individual motives, were the other socio-demographic variables was. Runners that were older (>46 years) are less likely to perceive individual motives to quit running than younger runners (<35 years) did (OR = 0.498; *p* < 0.001). Runners with higher education or who finished university, were more likely to quit running based on individual motives compared to runners with a lower of middle education (resp. OR = 2.012; *p* < 0.001 and OR = 2.721; *p* < 0.001). Similarly, to the model on social motives for quitting, runners with more than 5 years of running experience, were less likely (OR = 0.610; *p* < 0.05) to perceive individual motives to quit running compared to runners with less than 1 year of running experience. The running frequency was also found to be significant, those who run twice a week (OR = 0.654; *p* < 0.05) were less likely to perceive individual motives for quitting compared to runners who run once (or less) a week. Furthermore, runners who identify themselves with running (OR = 0.352; *p* < 0.001) were less to perceive individual motives to quit running. No significant differences were found for running context, and AIO-items running as a sport that is easy to practice and perceived advantages of running.

## 4. Discussion

The aim of this study was to gain insight among short-distance event runners into the perceived reasons to quit running, and to identify how these reasons are affected by socio-demographics (i.e., gender and age), running habits and AIOs on running. This is an important step toward expanding the evidence base to understand the reasons for dropout as perceived by runners. This is key to support runners in continued running and to address the barriers runners perceive adequately. The limitations of this study, such as the treatment of the data and its implications, are discussed at the end of the discussion section.

Our findings show that runners are more likely to perceive individual reasons to quit running than social reasons (Table 3). Physical constraints or injuries (item 7) is the most important reason to quit running, which is in line with previous studies [29,30,31,32,33], followed by being tired of running (item 6). Socials reasons to quit running because ‘my trainer is leaving’, or ‘my buddy quit running’ were less likely to be perceived as important. A possible explanation for this might be that a large group of the participants (approx. 60%) does not run in a social context but runs individually. This is in line with studies showing that running is an activity that is mostly practiced individually, outside the organized context of clubs [4,28,46]. For individual runners, individual reasons to quit running might be more applicable and easier to identify with, as compared to social reasons.

For individual reasons to quit running, significant differences were found for age, education level, experience with running, running frequency and one of the AIOs towards running; identification with running (Table 4). Furthermore, results showed that social reasons to quit running are significantly different depending on the gender, experience with running, running context and on the AIOs towards running; running as a sport that is easy to practice, perceived advantage of running and identification with running.

Compared to male runners, our results show that female runners perceive more social reasons to quit running. This result may be explained by the fact that women appear to attach greater value to social support [47,48,49]. A previous study by Vos et al. [19], in which a typology of female runners was constructed, did show that women valued connectedness with others. This finding was also reported by Pridgeon and Grogan [49], stating that loss of social support contributed to exercise dropout, especially among women. Another possible assumption would be that female runners, compared to male runners, run more often in a social context and therefore experience social reasons to quit running more often. However, this explanation is not supported by a previous study (N = 3727) on running typologies, which does not suggest that women are more likely to run in social contexts but often run in individual context as well [41]. Notably, in the present study, we did not found significant differences for individual reasons to quit running for gender. So, although female runners run in both social and individual contexts, social reasons to quit running are perceived more often by women than men.

Runners aged above 45 years, perceive fewer individual reasons to quit running as compared to younger runners below 35 years. This result might hint at the idea of people feeling more in control of their own time when ageing, as compared to having difficulties in seeking a way to incorporate running in their daily lives [47,48,50]. This might also be related to the fact that people over 45 are in a less exploratory phase of their lives, and thus do not perceive reasons to seek for different types of sports to practice [50]. Another explanation might be that these ‘older’ runners are more experienced and therefore, more aware of their bodies and potential injuries [30,51]. This is in line with a previous study, indicating that the most experienced runners included most runners being older than 45 [41]. What is notable is that there is no significant difference found for social reasons to quit running for age, indicating that reasons to quit from a social perspective are not dependent on age.

Our results suggest that runners who have a higher education or university degree perceive more individual reasons to quit running compared to runners with a low or middle educational degree. Runners with a university degree perceive these reasons about three times as much, and runners with a higher education twice as much. This is not the case for social reasons to quit running. The reason for this might be that runners with a higher or university degree have more trouble in finding a good work-life-sports balance, and thus have more trouble in prioritizing running on a day to day basis.

Running experience influenced both social and individual reasons to quit negatively, where runners who run for more than 5 years perceive less (social and individual) reasons to quit as compared to runners running for less than a year. We can hypothesize that runners who already have been running for more than 5 years have already been able to overcome obstacles and barriers (e.g., injuries or motivational loss) throughout the years and kept pursuing running [30,51]. On the other hand, participants running for less than a year might have a lower self-efficacy, i.e., confidence in one’s ability to overcome potential obstacles [52]. Another possible explanation is that experienced runners might feel more competent, and therefore are less afraid of getting injured or being dependent on external factors like a coach or a running group. A previous study, for instance, indicated that the more experienced runners (>7 years) were more likely to run “for the love of running” [25], which might indicate that regardless of some obstacles, their love for running helps them overcome these.

When looking at running frequency, the results suggest that runners who run twice a week perceive fewer individual reasons to quit running as compared to runners who run once a week or less. Notably, this is not the case for social reasons to quit running, nor for runners who run three times a week or more. Although these runners who run twice a week have a higher time investment compared to runners who run once a week or less, they might be able to better incorporate this activity in their schedule on a weekly basis [39]. For those running ≤1 per week, the involvement into running is lower, as compared to runners who dedicate to run twice a week. These ‘occasional’ runners might perceive more reasons to quit since they have not been able to commit to the sport that often on a training basis yet [39,41]. Additionally, a lower running frequency might also affect the feeling of competence or experience, which in turn might increase the fear of getting injured [30].

Although runners in our sample generally experienced more individual reasons to quit running, the running context positively influenced social reasons to quit running. Runners who run in a running group perceive more than three times as many social reasons to quit running compared to runners who run individual, and runners running at a running club more than four times as much. It might seem obvious that when one runs individually, fewer social reasons to quit can be observed. Interestingly though, individual runners do not perceive more individual reasons to quit running, as compared to social runners. Individual reasons to quit running might thus not be dependent on the running context but on other variables (e.g., age, running experience, running frequency) as stated in earlier studies [17,36,37].

Runners who do not think of running as a sport that is easy to practice, and do not perceive many advantages of running, perceive more social reasons to quit running. Instead of these advantages of running, these runners might value and need other AIOs (e.g., social support) to go running and therefore, experience more social reasons to quit running [49].

When one identifies as being a runner, our results indicate that this affects both social and individual reasons to quit running. Runners who identify themselves as a runner perceive more social reasons to quit running. This might indicate that runners who run in a social context (e.g., club or running group), identify themselves as being a ‘real’ runner and therefore might also depend more on their fellow runners (as a community) and social support. When for example a fellow runner quits, this might act as a trigger to quit running [49]. Contrary to this, runners who identify as being a runner perceive less individual reasons to quit running. A possible explanation might be that these are less likely to get tired of running, or running is their main sport. This is in line with previous studies indicating that runners who identify strongly with running are the more experienced, long-distance runners [41,43], hinting they might have been able to overcome these possible reasons to quit previously.

Based on our results, we argue that although we see significant differences related to gender in social reasons to quit running and significant ones related to age in individual reasons to quit running, these should not be considered conclusive. Our results showed that running characteristics (e.g., running experience, context, frequency, running AIOs) also influence one’s perceived reasons to quit running. We thus contribute to knowledge on running dropouts by drawing a more accurate picture of the situation.

### Limitations

Our study has some limitations. As part of our sampling strategy, we selected a subset of the dataset and included runners who participated in the 5 and 10 km distances of the running event. Through this, we purposively focused on novice and less experienced runners, who are more likely to drop-out. Although these runners might not be representative of all runners who perceive reasons to quit running, participants of large running events have been considered a representative selection of the broader recreational running community in previous studies [41,53].

In this study, we investigated runners’ perceived reasons to quit running. By asking perceived reasons, this study relies on self-reported data and the perception of the participants. We do not know if these reasons would be an actual reason to quit running. However, knowing more about the perception of runners might indicate possible solutions or interventions to lower drop-out rates.

Finally, some methodological limitations related to the dependent variables should be mentioned. As aforementioned, we had to recode our two dependent variables into binary variables because both scales were not normally distributed. We thus lost some information about individual differences. Yet, we were able to interpret the data relativity to the sample. Second, we used 7 items to construct the 2 independent variables. Next to these seven possible reasons to quit running, there are other reasons why runners may quit running. Here we decided to build further on previous studies and hence could benefit from items which have an acceptable internal consistency.

## 5. Conclusions

Our survey study shows that although gender and age have shown significant differences in perceived reasons to quit running, these should not be considered conclusive. Our findings implicate that running characteristics (e.g., running experience, context, frequency, running AIOs) also influence one’s perceived reasons to quit running. These insights could help policymakers to understand novice runners and their perceived reasons for a potential drop-out. This insight can be used to match public health policies to the motives and barriers of novice runners. Sports professionals (e.g., trainers and, coaches) could use this insight to lower drop-out rates among novice runners and eliminate potential perceived reasons to quit running.

## Figures and Tables

**Table 1:** Components including the number of items, Cronbach α, average scores and standard deviations.

Scale	Attitudes toward Running	Items	Cronbach α	N	Mean	SD
1	Perceived advantages of running	4	0.794	853	4.29	0.458
2	Identification with running	5	0.738	853	3.33	0.640
3	Running as a sport that is easy to practice	3	0.781	853	4.22	0.623
4	Social motives for quitting	3	0.941	853	1.79	0.722
5	Individual motives for quitting	4	0.712	853	3.33	0.784

**Table 2:** The descriptive statistics of the sample for the dependent and independent variables.

Variable	Measurement	n	%
Individual Motives Binary	Below	399	46.8
	Above	454	53.2
Social Motives Binary	Below	390	45.7
	Above	463	54.3
Gender	Male	387	47.8
	Female	422	52.2
Age	≤35 year	261	32.1
	36–45 year	239	29.4
	≥46 year	313	38.5
Education	Lower or middle education	273	33.5
	Higher education	332	40.8
	University	209	25.7
Experience	<1 years	248	29.2
	1–5 years	364	42.8
	>5 years	238	28.0
Running frequency	≤1x/week	384	45.1
	2x/week	350	41.1
	≥3x/week	117	13.7
Running context	Individual	526	61.8
	Friends, colleagues, small groups	226	26.6
	Clubs	99	11.6

**Table 3:** Mean scores, standard deviations, minimum and maximum values of the items.

Item No.	Item	Mean	SD	Min	Max
1	My running partners quit running ^1^	1.82	0.85	1	5
2	My running group falls apart ^1^	1.80	0.84	1	5
3	My trainer/coach is leaving ^1^	1.76	0.80	1	5
4	Preference for another sport ^2^	3.06	1.04	1	5
5	Reduction of leisure time ^2^	2.95	1.05	1	5
6	Tired of running ^2^	3.20	1.06	1	5
7	Physical constraints or injuries ^2^	4.14	0.77	1	5

Superscript number indicate to which scale, the items belong to. Social reasons to quit running indicated with a 1, and individual reasons indicated with 2.

**Table 4:** Results of the binary logistic regression, in odds ratios (Exp (β)) with regards to the reference group (ref.).

		Social Reasons (*n* = 803)	Individual Reasons (*n* = 803)
Constant		646,050 ***	42,827 ***
Gender	Male	Ref.	Ref.
	Female	1.642 **	1.234
Age	≤35 year	Ref.	Ref.
	36–45 year	1.018	0.777
	≥46 year	1.402	0.498 ***
Education	Lower or middle education	Ref.	Ref. ***
	Higher education	1.193	2.012 ***
	University	0.972	2.721 ***
Experience	<1 years	Ref.	Ref.
	1–5 years	0.829	0.888
	>5 years	0.610 *	0.610 *
Running frequency	≤1x/week	Ref.	Ref.
	2x/week	0.717	0.654 *
	≥3x/week	0.734	0.799
Running context	Individual	Ref. ***	Ref.
	Friends, colleagues, small groups	3.352 ***	1.203
	Clubs	4.541 ***	1.361
AIO toward running	Running as a sport that is easy to practice	0.502 ***	0.985
	Perceived advantages of running	0.314 ***	0.992
	Identification	1.366 *	0.352 ***
Nagelkerke R^2^		0.278	0.244

* = *p* < 0.05; ** = *p* < 0.01; *** = *p* < 0.001.

## Supplementary Materials

The following are available online at https://www.mdpi.com/1660-4601/17/17/6046/s1, File S1: Figure of Descriptive Statistics and File S2: Mean scores and SD of the Items.
