# Supplementary figures and images for: Runner’s Perceptions of Reasons to Quit Running: Influence of Gender, Age and Running-Related Characteristics
Source: Int J Environ Res Public Health. 2020 Aug 20;17(17):6046. doi: 10.3390/ijerph17176046 (PMC7503581; doi:10.3390/ijerph17176046)

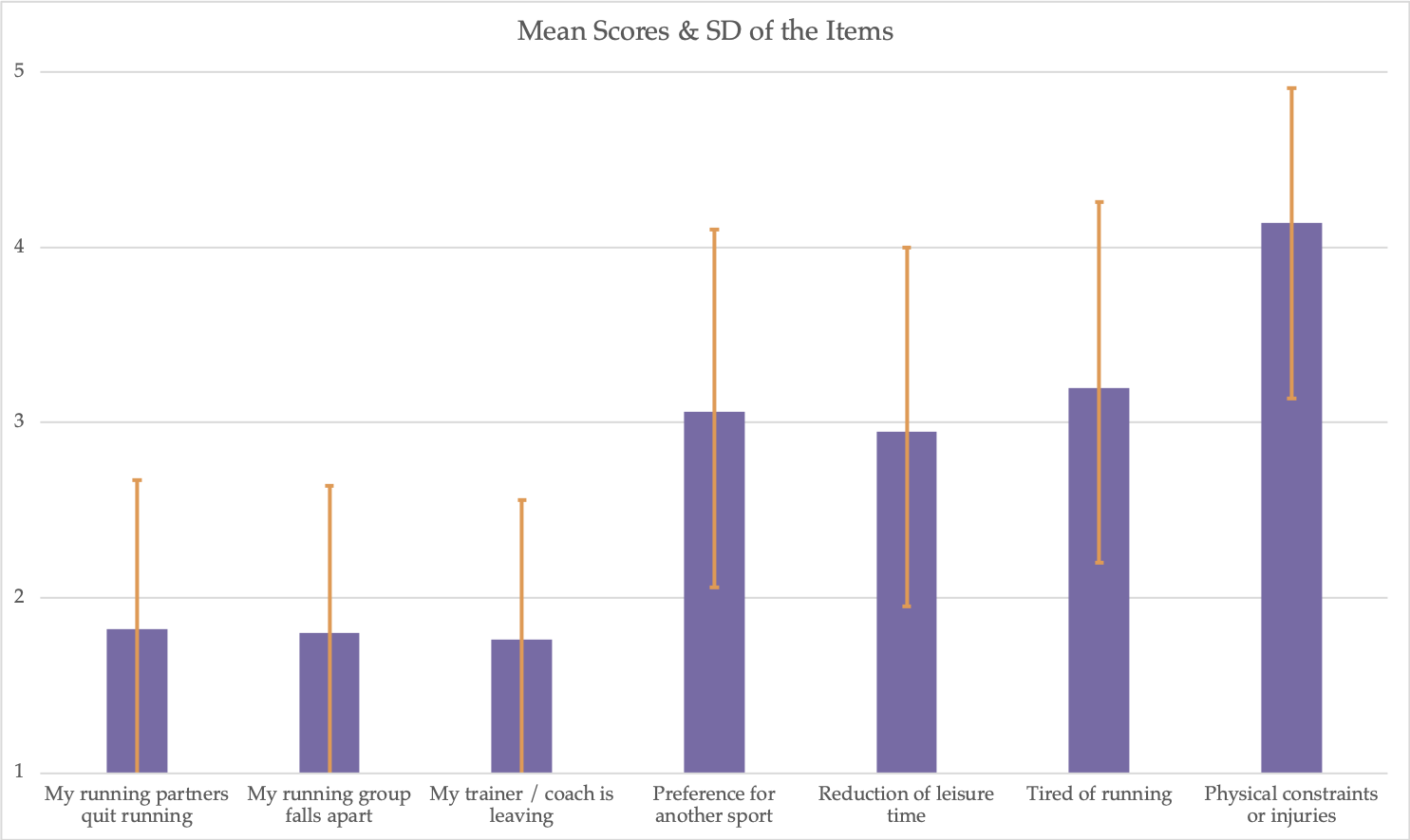

Supplement: Supplementary file 1 [file ijerph-17-06046-s001.zip › Supplementary Files/S2 Items Mean and SD.png]

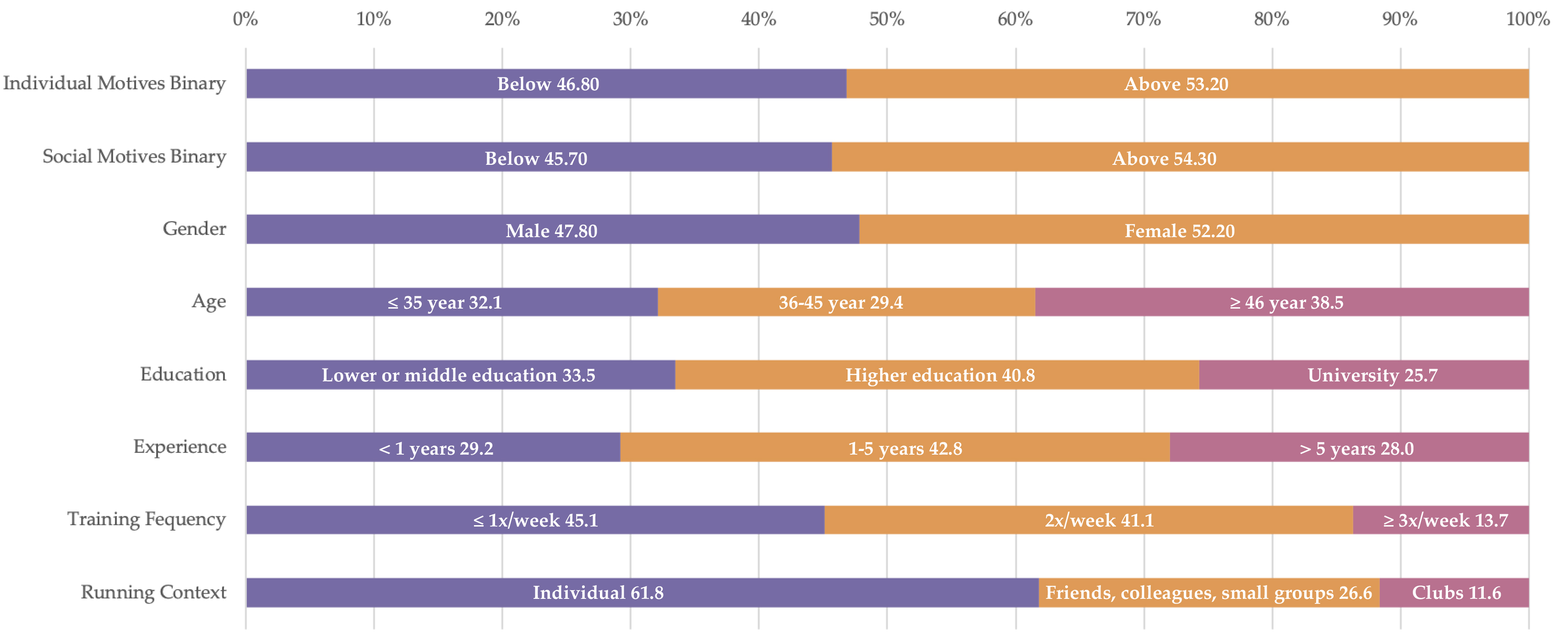

Supplement: Supplementary file 1 [file ijerph-17-06046-s001.zip › Supplementary Files/S1 Descriptive Statistics.jpg]
